# Serum Anti-BPAG1 Auto-Antibody Is a Novel Marker for Human Melanoma

**DOI:** 10.1371/journal.pone.0010566

**Published:** 2010-05-10

**Authors:** Takashi Shimbo, Atsushi Tanemura, Takehiko Yamazaki, Katsuto Tamai, Ichiro Katayama, Yasufumi Kaneda

**Affiliations:** 1 Division of Gene Therapy Science, Osaka University Graduate School of Medicine, Osaka, Japan; 2 Department of Dermatology, Osaka University Graduate School of Medicine, Osaka, Japan; Dana-Farber Cancer Institute, United States of America

## Abstract

Malignant melanoma is one of the most aggressive types of tumor. Because malignant melanoma is difficult to treat once it has metastasized, early detection and treatment are essential. The search for reliable biomarkers of early-stage melanoma, therefore, has received much attention. By using a novel method of screening tumor antigens and their auto-antibodies, we identified bullous pemphigoid antigen 1 (BPAG1) as a melanoma antigen recognized by its auto-antibody. BPAG1 is an auto-antigen in the skin disease bullous pemphigoid (BP) and anti-BPAG1 auto-antibodies are detectable in sera from BP patients and are used for BP diagnosis. However, BPAG1 has been viewed as predominantly a keratinocyte-associated protein and a relationship between BPAG1 expression and melanoma has not been previously reported. In the present study, we show that bpag1 is expressed in the mouse F10 melanoma cell line *in vitro* and F10 melanoma tumors *in vivo* and that BPAG1 is expressed in human melanoma cell lines (A375 and G361) and normal human melanocytes. Moreover, the levels of anti-BPAG1 auto-antibodies in the sera of melanoma patients were significantly higher than in the sera of healthy volunteers (p<0.01). Furthermore, anti-BPAG1 auto-antibodies were detected in melanoma patients at both early and advanced stages of disease. Here, we report anti-BPAG1 auto-antibodies as a promising marker for the diagnosis of melanoma, and we discuss the significance of the detection of such auto-antibodies in cancer biology and patients.

## Introduction

Melanoma is one of the most aggressive tumors due to its strong capacity to metastasize. In the United States, there were an estimated 62,480 new melanoma cases and 8,420 deaths caused by melanomas in 2008 [1]. Although the 5-year survival rate of patients with early stage localized melanoma is greater than 90%, survival rates drop to less than 20% once the melanoma has metastasized to distant sites [1]. In general, early diagnosis of cancers greatly improves the survival of patients. Therefore, great efforts have been made to screen tumor markers for early diagnosis. Several melanoma markers (e.g. gp100, MART-1 and tyrosinase) have been detected and proposed for immunotherapy approaches [2], [3], [4]. With regards to melanoma markers in serum, S100 protein, 5-S-cysteinyldopa and 6-hydroxy-5-methoxyindole-2-carboxylic acid can be useful although levels tend to be more up-regulated in advanced melanomas. As such, these particular markers are not suitable for the early detection of malignant melanoma [5]. Glypican-3 (GPC3), however, is overexpressed in melanoma and its serum concentration can serve as an early stage melanoma diagnostic marker [6], [7]. Nevertheless, from a practical prospective, use of only one biomarker may lack sensitivity and specificity and diminish clinicopathologic value. The availability of multiple markers would make the diagnosis of melanoma more reliable, and thus there is a need to identify and assess additional melanoma markers.

In the present study, we developed a screening method to detect tumor markers recognized by auto-antibodies to these proteins in serum. Using this method, we found that bullous pemphigoid antigen 1 (BPAG1) was expressed in both melanoma cell lines and normal melanocytes. BPAG1 is a plakin family protein that anchors keratin filaments to hemidesmosomes [8]. Another protein BPAG2, a transmembranous collagen, is also expressed in the skin and is a component of hemidesmosomes [8]. Deletion of the *dst* gene, that encodes bpag1, disrupts hemidesmosomes structure, resulting in the failure of hemidesmosomes to associate with keratin filaments [9]. Both BPAG1 and BPAG2 can serve as auto-antigens in bullous pemphigoid (BP) [10], [11], [12]. Auto-antibodies to BPAG1 and BPAG2 maybe detected in the sera of BP patients, and assessment of antibody levels can be used for BP diagnosis and clinical management. While passive transfer experiments have shown that BPAG2 antibodies have pathogenic relevance to BP, the clinicopathological significance of BPAG1 antibodies, has not yet been fully elucidated [13]. It has been hypothesized that anti-BPAG1 auto-antibodies might interfere with hemidesmosome integrity, but this has not been proven [9].

Here, we show that the level of auto-antibodies against BPAG1 in the sera of melanoma patients, at both early and advanced stages, was significantly higher than levels in the sera of healthy volunteers. These findings identify anti-BPAG1 auto-antibodies as a novel and promising tumor biomarker in the detection of melanoma.

## Materials and Methods

### Libraries, bacteria and helper phage

The human single-fold scFv libraries I + J (Tomlinson I + J), *E. coli* TG1 and HB2151, and KM13 helper phage were all kindly provided by the Medical Research Council (MRC). The scFv library was prepared as previously described [14]. The scFv library was cloned into the pIT2 expression vector, which contains a lac promoter and a pelB leader sequence upstream of the VH-(G_4_S)_3_-VL insert; the insert is followed by 6×His and myc tags, an amber stop codon and the gene encoding the pIII phage coat protein. Thus, in a suitable non-suppressor strain (HB2151), addition of isopropyl­thio-β-D-galactoside (IPTG) induces only scFv and not scFv–pIII fusion expression.

### Mice

Female C57BL/6N mice (6 weeks old) were studied (Charles River Laboratories Japan, Inc., Japan) Animal experiments were performed in accordance with the guidelines of the Osaka University Graduate School of Medicine.

### Cell lines and culture

Mouse melanoma cell line F10, mouse fibroblast cell line NIH-3T3, human melanoma cell lines A375, G361 and human epidermoid carcinoma A431 were obtained from the American Type Culture Collection (ATCC, USA). Normal human keratinocyte (NHK) cells and melanocyte (NHM) cells were obtained from Lonza (USA). F10, NIH-3T3, A375, G361 and A431 cells were maintained in Dulbecco's modified Eagle's medium (DMEM) (Nacalai Tesque Inc., Japan). DMEM was supplemented with 10% fetal bovine serum (FBS) (Biowest, France), 100 units/ml penicillin and 0.1 mg/ml streptomycin (penicillin-streptomycin mixed solution) (Nacalai). NHK and NHM cells were maintained in keratinocyte growth medium (KGM) (Lonza) and melanocyte growth medium (MGM-4) (Lonza), respectively.

### Preparation of tumor lysates and the isolation of sera from tumor-bearing mice

F10 cells (5×10^6^) were intradermally injected into the backs of C57BL/6N mice. After 4 weeks, tumors were excised, and protein was extracted using T-PER Tissue Protein Extraction Reagent (Pierce, USA), according to the manufacturer's instructions. At the same time as the tumor excision, whole blood was collected, and the sera were isolated using Capiject Capillary Blood Collection Tubes (Terumo Corp., Japan).

### *In vivo* screening of tumor-homing phages and isolation of the tumor-binding scFv

F10 cells (5×10^6^) were intradermally injected into the backs of C57BL/6N mice. After tumors reached 7 to 8 mm in diameter, we injected the phage library (1×10^13^ CFU) dissolved in 100 µl saline into the tail veins of tumor-bearing mice. After 15 min, the animals were sacrificed by an overdose of anesthetic and perfused via the heart with 100 ml of PBS [15]. Next, the tumor tissue was snap-frozen in liquid nitrogen and homogenized in a mortar. Tumor-homing phages were eluted by using 500 µl of 0.1 M glycine (pH 2.2) for 15 min, and then the solution was neutralized with 50 µl of 2 M Tris-HCl (pH 8). Next, 50 µL of Protein A (GE Healthcare, USA) was added to the neutralized phage solution, and the mixture was rotated for 1.5 h at 4°C. After washing with PBS to remove unbound phages, phages bound to protein A were used to infect log-phase HB2151 for 1 h at 37°C; then, the cells were plated on 2×YT agar plates containing 100 µg/ml carbenicillin. After 16 h of incubation at 37°C, a nitrocellulose membrane soaked in 100 mM IPTG (Takara Bio Inc., Japan) for 10 min was placed on the 2×YT plate for 4 h at 37°C. The plate was stored at 4°C as a master plate for *E.coli* recovery. The membrane was washed 3 times with PBS containing 0.1% tween 20 and blocked 30 min with PBS containing 5% skim milk (Nacalai). Then, the membrane was incubated with tumor lysate followed by tumor-bearing mouse serum. The complexes of auto-antibodies bound to tumor proteins were detected with the anti-mouse IgG horseradish peroxidase-conjugated antibody (GE Healthcare) followed by ECL Western Blotting Kit (GE Healthcare).

### Identification of tumor antigen with MALDI-TOF mass spectrometry

Positive clones that were recovered from the stored 2×YT agar plate were re-plated onto a fresh 2×YT agar plate and incubated for 16 h at 37°C. Next, a nitrocellulose membrane soaked with 100 mM IPTG was placed onto the 2×YT agar plate for 4 h at 37°C. After washing with PBS containing 0.1% tween 20, the membrane was blocked 30 min with 0.5% polyvinylpyrrolidone K30 (Nacalai), and then the membrane was incubated with tumor lysate. The scFv-tumor protein complexes were stained with 0.5% ponceau (Wako Pure Chemical Industries, Ltd., Japan)/5% acetic acid in distilled water, and the membrane around the area was excised. The protein on the excised membrane was digested with 100 ng/ml trypsin (Sigma-Aldrich, USA)/40 mM ammonium bicarbonate (Nacalai) for 6 h at 37°C. Then, the solution was dried up, and saturated α-cyano-4-hydroxy cinnamic acid (Wako), 1% trifluoroacetic acid, and 50% acetonitrile was added. After desalting with a ZipTip C18, the solution was analyzed using an Ultraflex MALDI-TOF/TOF instrument (Bruker Daltonik, Germany). The mass spectrometry data were analyzed with the Mascot search engine (http://www.matrixscience.com).

### RT-PCR and real-time PCR

RNA was extracted using Isogen (Nippon Gene, Japan), and 1 µg of total RNA was converted to cDNA with SuperScript III (Invitrogen, USA), according to the manufacturer's instructions. Mouse bullous pemphigoid antigen 1 (bpag1), TBC1 domain family member 13 (tbc1d13), uncharacterized protein C7orf30 homolog (c7orf30), and β-actin were amplified using SYBR Premix Ex Taq (Takara Bio) and an ABI Prism 7900 sequence detector (Applied Biosystems, USA). Human BPAG1, BPAG2, and β-actin were amplified with TaKaRa Ex Taq Hot Start Version, and PCR products were analyzed by electrophoresis on 1% agarose gels. All procedures were performed according to the manufacturer's instructions.

The primers were as follows:

Mouse bpag1-f:5′- TTGGAACAGACCTGGAGACC-3′

Mouse bpag1-r:5′- GTTCAGCCTTTCCATTTCCA-3′

Mouse tbc1d13-f:5′- AGGCCAACATGGGTGTATTC -3′

Mouse tbc1d13-r:5′- AGGGTTTGGGTTCAGAGGAT-3′

Mouse c7orf30-f:5′- GAGGGGAAGGACGCTGAC -3′

Mouse c7orf30-r:5′- TGGAAGCATCAAATGGATCA-3′

Mouse β-actin-f:5′- CCACTGCCGCATCCTCTTCC-3′

Mouse β-actin-r:5′- CTCGTTGCCAATAGTGATGACCTG -3′

Human BPAG1-f:5′- CCAGCCCGGTTAACTATTGA -3′

Human BPAG1-r:5′- TGGCAGAGCTGTAAGATCCA-3′

Human BPAG2-f:5′- GCTGGAGATCTGGATTACAATGA-3′

Human BPAG2-r:5′- CCTTGCAGTAGGCCCTGA-3′

Human β-actin -f:5′- GAGCTACGAGCTGCCTGACG-3′

Human β-actin -r:5′- GTAGTTTCGTGGATGCCACAG-3′

### Detection of BPAG1 by Immunoprecipitation (IP) and Western blotting

Cells (1×10^7^) were trypsinized, washed in cold PBS and resuspended in 500 µl RIPA buffer. IP of total cell lysates were incubated with anti-BPAG1 antibody (sc-13776) (Santa Cruz Biotechnology Inc., USA) or normal goat IgG (Santa Cruz) for 1 h followed by protein G agarose (GE Healthcare) overnight. SDS-PAGE and Western blotting were performed, as previously described [16] with anti-BPAG1 antibody. After incubation with HRP conjugated anti-goat IgG (R&D systems, USA), signals were detected with ECL Western Blotting Detection Regents (GE Healthcare) according to the manufacturer's instructions.

### Quantification of BPAG1 auto-antibodies in sera

Approval for this study was obtained from the Institutional Review Board of the Osaka University Hospital (#08312). Written informed consent was obtained from all participants before the study. We collected sera from 55 melanoma patients and 27 healthy volunteers. The malignant melanoma patients studied here consisted of 24 men and 31 women with an average age of 62.6 years (range, 22 to 86 years); 13 had stage 0 (*in situ*) or stage I; 5 had stage II; 11 had stage III; 26 had stage IV. The healthy volunteers consisted of 15 men and 12 women with an average age of 31.6 years (range, 24 to 49years). The sera samples were stored at −30°C prior to use and the serum levels of anti-BPAG1 auto-antibodies were determined using a BP230 ELISA kit (MBL, Japan) according to the manufacturer's instructions. The INDEX was calculated as follows; INDEX  =  (sample absorbance – negative control absorbance [INDEX = 0])/(positive control absorbance [INDEX = 100] - negative control absorbance) ×100 [17]. The Mann-Whitney U test was used to determine statistical significance, and p<0.05 was considered statistically significant.

## Results

### Identification of bpag1 as a tumor antigen recognized by auto-antibodies

**Figure 1:**
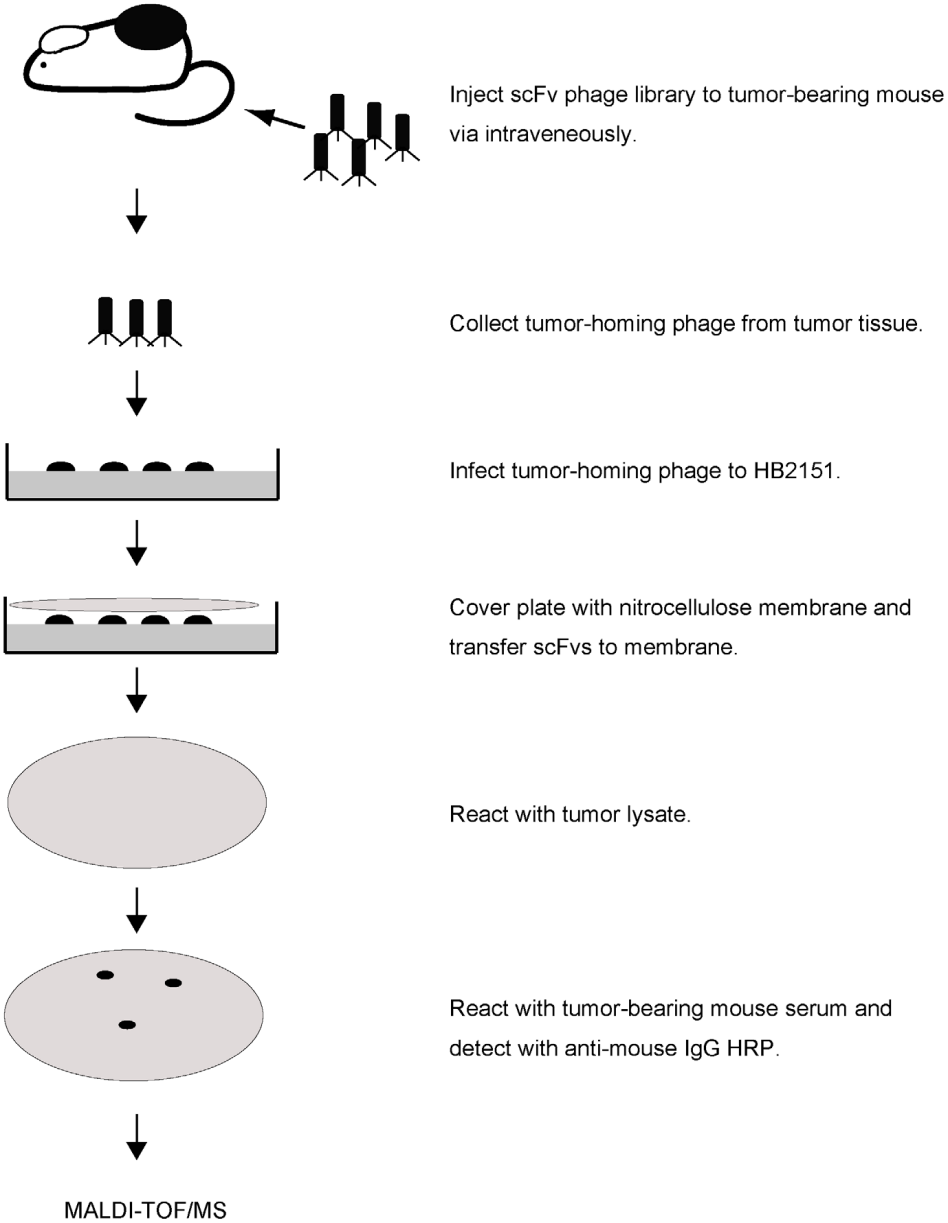
Overview of the rapid method for isolating auto-antibody against tumor-associated antigen (TAA) using a scFv library. The tumor-homing scFv-presenting phages were collected from tumors that were injected with a scFv library. The collected phages were infected to HB2151 for scFv secretion. The secreted scFvs from HB2151 were transferred to nitrocellulose membranes by colony lift. The membranes were incubated with tumor lysate followed by serum from a tumor-bearing mouse. The scFv-tumor protein complex was detected by auto-antibodies. The complex was digested into peptide by trypsin and analyzed using MALDI-TOF mass spectrometry for identification.

**Figure 2:**
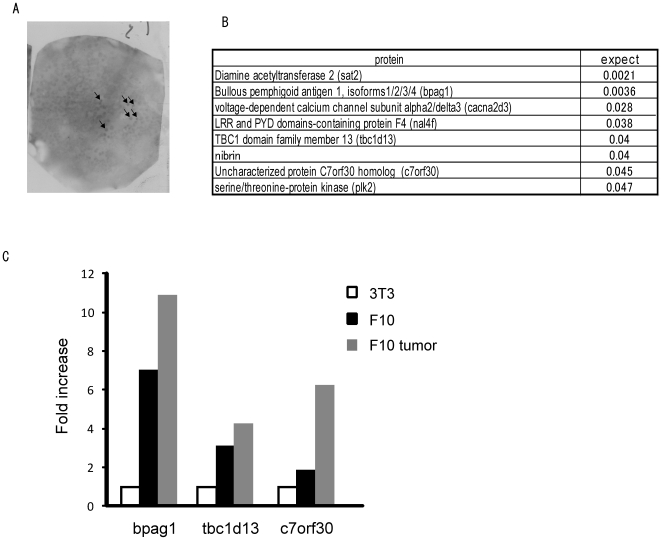
Identification of bpag1 as a tumor antigen recognized by auto-antibodies. (A) An example of the screening output. ScFv-tumor antigen complex was detected with auto-antibodies in tumor-bearing mouse serum. (B) Eight candidates were identified by MALDI-TOF mass spectrometry with statistical significance (p<0.05); expect  =  expectation value. (C) Comparison of bpag1, tbc1d13 and c7orf30 expression in NIH-3T3 cells (white bar), F10 melanoma cells (black bar) and F10 melanoma tumors (grey bar) by SYBR Green real-time PCR.

An overview of the screening procedure is shown in Figure 1. We performed the screening on mouse F10 melanoma cell lines. The tumor-homing phages were collected by *in vivo* biopanning in tumor-bearing mice. The phages were used to infect HB2151 for scFv expression and plated on a 2×YT agar plate. After 16 h, scFv expression was induced with IPTG, and the resulting scFvs were transferred onto a nitrocellulose membrane. The membrane was incubated with F10 melanoma tumor lysate followed by F10 tumor-bearing mouse serum. The scFv-tumor protein complexes on the nitrocellulose membrane were detected with the auto-antibodies in the serum collected from the tumor-bearing mice (Figure 2A). We performed the screening procedure several times and selected high-signal clones, distinct from background signals, for further experiments. Next, we analyzed the proteins that were detected with auto-antibodies by performing MALDI-TOF mass spectrometry, and we identified 8 potential melanoma marker candidates with statistical significance (p<0.05) (Figure 2B). The candidates were ordered according to the expectation value, which is the expected frequency of the matches to be obtained from mass spectrometry merely by chance. We compared the expression of the 8 candidates among NIH-3T3 cells, F10 melanoma cells and F10 melanoma tumors by real-time PCR. Bpag1, tbc1d13 and c7orf30 were expressed at much higher levels in F10 melanoma cells (7.0-, 3.1- and 1.9-fold, respectively) and F10 melanoma tumors (10.9-, 4.2- and 6.3-fold, respectively) as compared to NIH-3T3 cells (Figure 2C); the expression levels of the remaining 5 candidates in melanoma cells and tumors were less than the expression level in NIH-3T3 cells (data not shown). Among the three candidates, we selected bpag1 for further investigation because bpag1 was expressed most abundantly in F10 melanoma cells and tumors, and is known to have a restricted tissue expression pattern, including skin, brain and muscle [18].

### Differential expression of BPAG1 and BPAG2 in normal human melanocytes and human melanoma cell lines

**Figure 3:**
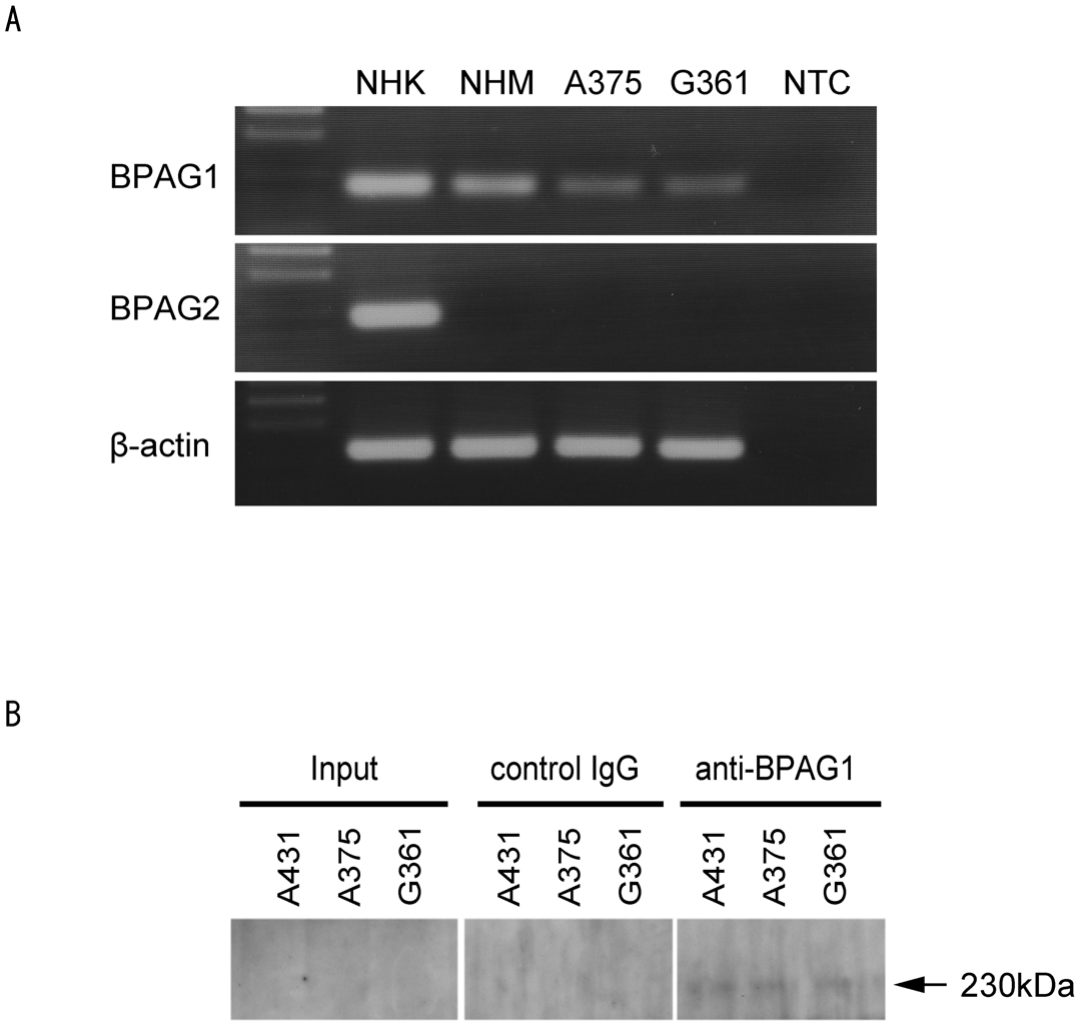
Expression of BPAG1 in normal human melanocytes and human melanoma cell lines. (A) The expression of BPAG1 and BPAG2 mRNA was quantified by RT-PCR in normal human melanocytes (NHM) and human melanoma cell lines A375 and G361. Normal human keratinocyte (NHK) mRNA was used as a positive control. β-actin was amplified as a loading control for cDNA. NTC; no template control. (B) The expression of BPAG1 protein was detected by IP-western blotting in human melanoma cell lines A375 and G361. A431 was used as positive control for BPAG1. The arrow indicates BPAG1.

We used RT-PCR to confirm the expression of BPAG1 in human melanomas. BPAG1 is expressed in normal human keratinocytes and is a component of hemidesmosomes along with BPAG2. Thus, we used normal human keratinocytes as a positive control for both these proteins. We identified BPAG1 expression in human melanoma cell lines (A375 and G361) and normal human melanocytes (Figure 3A). However, we did not detect BPAG2 expression in the human melanoma cell lines or normal human melanocytes (Figure 3A). We also detected BPAG1 protein in A375 and G361 by IP-western blotting (Figure 3B).

### Quantification of anti-BPAG1 auto-antibodies in sera

**Figure 4:**
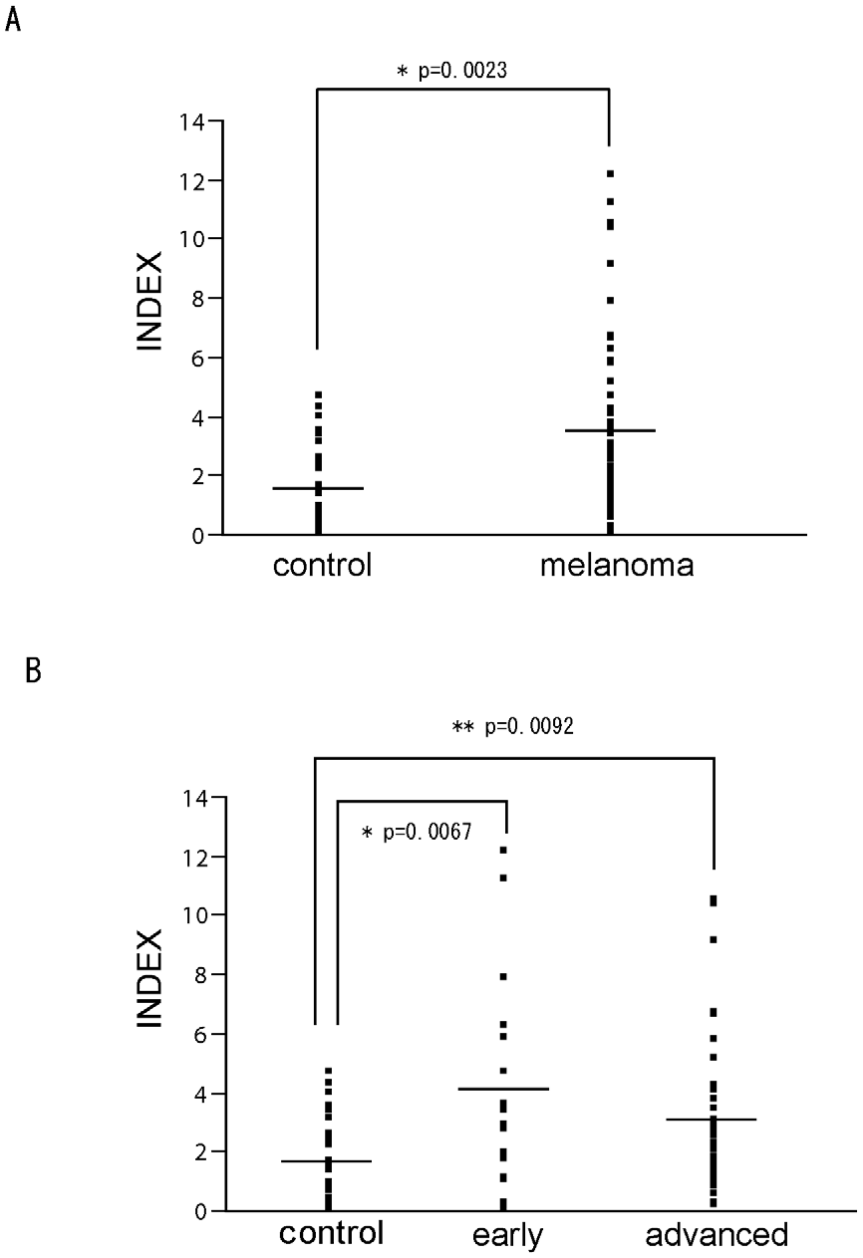
Quantification of anti-BPAG1 auto-antibodies in melanoma patients. (A) The levels of anti-BPAG1 auto-antibodies in sera collected from healthy volunteers and melanoma patients were quantified using a MESACUP BP230 ELISA Kit. The INDEX values were plotted and the average INDEX values as shown (±S.E.M.) for control subjects (1.64±0.27) and melanoma patients (3.47±0.40). (B) The melanoma patients were classified using the American Joint Committee on Cancer (AJCC) 2002 staging criteria. *In situ*, stage I or stage II patients were categorized as “early”, while stage III or stage IV patients were categorized as “advanced”. The average INDEX values (±S.E.M.) of early and advanced melanoma patients were 4.14±0.83 and 3.15±0.43, respectively; the bars indicate the average INDEX value.

Auto-antibodies against BPAG1 are found in sera from BP and therefore BPAG1 maybe a highly immunogenic protein. We hypothesized that the human immune system might generate auto-antibodies against BPAG1 expressed in human melanomas. To assess this possibility, we collected sera from 55 melanoma patients and 27 healthy volunteers and quantified the serum levels of anti-BPAG1 auto-antibodies by ELISA (Figure 4A). The average (±S.E.M.) INDEX value of the control group and the melanoma group was 1.64 (±0.27) and 3.47 (±0.40), respectively. We classified melanoma patients as follows: *in situ*, stage I or stage II patients were “early” (n = 18), and stage III or stage IV patients were “advanced” (n = 37). The levels of anti-BPAG1 auto-antibodies were much higher in both early and advanced stage melanoma patients (p<0.01) as compared to the healthy volunteers (Figure 4B). The average INDEX value (±S.E.M.) for the early and advanced melanoma patients was 4.14 (±0.83) and 3.15 (±0.43), respectively. Next, we evaluated the possibility of using anti-BPAG1 auto-antibodies as a melanoma detection marker. The maximum INDEX value in healthy volunteers (4.64) was defined as the cut off level. Applying these criteria, the positive rates of serum anti-BPAG1 auto-antibodies were 23.6% in total melanoma patients (13/55), 33.3% in early stage melanoma patients (6/18), and 18.9% in advanced stage melanoma patients (7/37) (Table 1). We also conducted indirect immunofluorescence studies using 4 melanoma patients' sera and demonstrated that IgG antibodies produced in patients reacted with skin basement membrane zone with variability in staining intensive (down to a dilution of 1∶160).

**Table 1:** Positive rates of serum anti-BPAG1 auto-antibody in stage-classified melanoma patients.

stage	total sample number	BPAG1 auto-antibody positive	%
early	18	6	33.3
advanced	37	7	18.9
total	55	13	23.6

The maximum INDEX value in healthy volunteers (4.64) was defined as the cut off level. The patients were classified by using the American Joint Committee on Cancer (AJCC) 2002 staging criteria. *In situ*, stage I or stage II patients were classified as “early”, while stage III or stage IV patients were classified as “advanced”.

## Discussion

In the present study, we developed a novel screening method for detecting tumor biomarkers. Our method is similar to SEREX in that we use serum containing anti-tumor auto-antibodies, but unlike SEREX, however, our method does not require a cDNA library from tumor tissue [19], [20], [21]. We anticipate that our method has clinical applicability, because it can be applied to human patients by modifying the method to select tumor-homing phage. In this report, we selected tumor-homing phage using tumor-bearing mice, but it is also possible to use tumor specimens [22]. Moreover, the phage screening could be conducted in human cancer patients without any detectable toxicity [23]. This method therefore has the potential to identify the most suitable tumor antigens for diagnosis or vaccination.

Nevertheless, our method did not detect previously identified melanoma antigens, such as gp100, tyrosinase, TRP-1 and TRP-2; instead, it detected completely different proteins. Gp100, tyrosinase, and TRP-2 were identified previously as melanoma antigens recognized by cytotoxic T cells from cancer patients, and TRP-1 was identified as a melanoma antigen recognized by IgG antibodies in the serum of a melanoma patient [24]. The epitopes of these antigens can efficiently activate tumor immunity, and they have been developed for cancer immunotherapy trials [25]. Although auto-antibodies to these melanoma antigens were detected in the sera of melanoma patients, the antigens were not frequently identified in sera from melanoma patients [26], [27], [28]. A possible explanation might be that auto-antibodies to such melanoma antigens exist in the sera of melanoma patients, but lack sensitivity or are present at low titers. Proteins identified by our screening method, however, have the potential to elicit the production of auto-antibodies in tumor-bearing individuals. In other words, our screening method may detect highly immunogenic proteins. Thus, it can be an effective tool for identifying detection markers in serum.

Given the differences in immune systems between mice and human, it is possible that the positive results screened in mice might be negative in humans. Ideally, screening procedures for auto-antibodies need to be performed in human melanoma patients. However, although the phage screening has been conducted in human cancer patients without any detectable toxicity, the method is still at a clinical trial stage [23]. It is therefore not yet straightforward or practicable to conduct phage screening in humans. With regards to our studies, screening mice also has the advantages of repeatability and uniformity of samples. On the other hand, it is difficult to conduct repeated screening in human cancer patients. Moreover, since the background of all the murine samples is uniform in our screening, any consistently positive result from repeated screenings will be highly reliable as tumor markers. In contrast, since the tumor stages and immunological states of patients are diverse, the results of screening using human patient sera would need extensive re-validation work.

We also showed that BPAG1 is expressed in human melanoma cell lines and that auto-antibodies against BPAG1 can be a potent melanoma marker. BPAG1 is expressed in normal keratinocytes within hemidesmosomes in association with BPAG2 and other proteins [8]. We did not detect any BPAG2 expression in human melanoma cell lines and melanocytes, and thus BPAG1 may have distinct functions in melanomas and melanocytes. BPAG1 is also expressed in some neurons and is involved in axonal neurofilament aggregation and axonal microtubule disorganization [29], [30]. The expression of BPAG1 in a human epidermoid carcinoma cell line (A431) and mammary ductal carcinoma *in situ* has also been confirmed [31], [32]. Thus BPAG1 may have an as yet undefined role related to tumorigenesis or tumor progression; overexpression and suppression of BPAG1 in melanocytes and melanomas will be necessary to determine this in more detail.

Some patients with melanoma develop vitiligo-like white patches, known as melanoma-associated hypopigmentation (MAH), on their skin [33]. Interestingly, the presence of vitiligo in melanoma patients may correlate with improved prognosis [34], [35]. Such patients with vitiligo could have more effective immunity against melanoma than patients without vitiligo. In general, the appearance of autoimmune phenomena improves the outcome of cancer patients [36]. However, such autoimmune phenomena are suppressed in most cancer patients by regulatory T (Treg) cells [37]. Treg cells inhibit CD8^+^ and CD4^+^ T cells, which are major components of cancer immunosuppression [38], [39]. The depletion of Treg cells from tumor-bearing mice promotes tumor regression [38], [39]. Interestingly, auto-antibodies were detected in mice showing tumor regression [38]. The efficiency of Treg-cell depletion may correlate with the emergence of auto-antibodies. Thus, the presence of anti-BPAG1 auto-antibodies may correlate with the occurrence of autoimmune responses in melanoma patients. By quantification of anti-BPAG1 auto-antibodies, it maybe possible to predict the immune status of cancer patients. In theory, immunotherapy against melanoma, therefore, might be more effective for patients with anti-BPAG1 auto-antibodies than those without antibodies.

We showed that BPAG1 is expressed both in melanocytes and melanomas. Since BPAG1 expression is maintained throughout the stages of melanoma tumorigenesis, BPAG1 auto-antibodies can be created even in the early stage of melanoma. We showed that the levels of anti-BPAG1 auto-antibodies are higher in melanoma patients at both early and advanced stages than in healthy volunteers (Figure 4 and Table 1). This result suggests that anti-BPAG1 auto-antibodies can be present in early stage melanoma, i.e. before it metastasizes. Thus, anti-BPAG1 auto-antibodies have the potential to be a promising melanoma biomarker. To test this hypothesis, we are planning to conduct a larger clinical study.

To the best of our knowledge, this report is the first to show BPAG1 expression in human melanomas and melanocytes and to highlight the potential of anti-BPAG1 auto-antibodies in the serum .as a marker of melanoma. We also demonstrated the effectiveness of our screening method. We will continue to develop this method into a more sophisticated method that can be used clinically. To validate the use of anti-BPAG1 auto-antibodies for melanoma diagnosis, we need to clarify the function of BPAG1 in melanoma and the mechanism of auto-antibody creation. These studies will be fundamental in assessing whether BPAG1 auto-antibodies can have an impact on improving the prognosis of melanomas.
